# Expression of 5-hydroxytryptamine 7 receptor in intestinal mucosa correlates with the degree of intestinal inflammation in Crohn’s disease

**DOI:** 10.1186/s12876-022-02513-5

**Published:** 2022-11-15

**Authors:** Zhangwei Xu, Jason J. Chen, Qiao Mei, Yang Li, Jianming Xu

**Affiliations:** 1 Department of Gastroenterology, First Affiliated Hospital of Anhui Medical University, 230022 Hefei, Anhui China; 2 Department of Pathology and Cell Biology, Columbia University, Vagelos College of Physicians and Surgeons, 10032 New York, NY USA

**Keywords:** 5-hydroxytryptamine 7 receptor, Crohn’s disease, Intestinal inflammation, Magnetic resonance imaging, Colonoscopy

## Abstract

**Background:**

Crohn’s disease (CD), an inflammatory bowel disease (IBD), is a complex and heterogeneous disease characterized by nonspecific transmural inflammation of the gastrointestinal tract. CD has a variety of potential causes with no effective treatment available yet. Current clinical laboratory findings from patients do not provide direct indication of the status of mucosal inflammation in the intestine. Recently, it has been found that intestinal inflammation is generally associated with increased levels of 5-hydroxytryptamine (5-HT), which acts as an important gastrointestinal signaling molecule in intestinal homeostasis by stimulating specific receptors. Most previous researches were carried out in vitro or with animal models, and there was a lack of authentic clinical research. In this study, clinical specimens from patients with Crohn’s disease were used to investigate the expression of 5-hydroxytryptamine 7 receptor (5-HT7R) in the induction and development of chronic non-specific inflammatory bowel disease.

**Methods:**

Patients with CD admitted to the Department of Gastroenterology in the First Affiliated Hospital of Anhui Medical University between June 2014 and January 2018 were recruited, among which 28 were in active disease and 32 were in remission. In addition, 20 patients who had no obvious abnormality by colonoscopy in the hospital during the same time period were recruited into the control group. Data of clinical disease activity (CDAI), CD endoscopic score (SES-CD) and magnetic resonance score (MaRIA) were collected from those two groups of patients. The expression and distribution of 5-HT7R were investigated and their correlations with clinical CDAI, MaRIA, and endoscopic SES-CD scores were analyzed.

**Results:**

Our study demonstrated that 5-HT7R is expressed in intestinal neurons and CD11_C_-positive cells in human colon. In CD11c/CD86 double-positive cells in the bowel, 5-HT7R expression was significantly increased in the inflammatory area in the bowel of CD patients, and it was closely related to disease severity, MaRIA, and SES-CD scores.

**Conclusion:**

The expression of 5-HT7R was significantly correlated with the degree of gut inflammation in CD patients and could be a potential biomarker for disease activity and the therapeutic efficacy in patients with Crohn’s Disease.

## Introduction

Crohn’s disease (CD), an inflammatory bowel disease (IBD), is a complex and heterogeneous disease characterized by chronic, recurrent episodes of gastrointestinal inflammation and nonspecific wall penetrating inflammation. The potential pathogenic causes of CD include heredity, immunity, environment and microorganisms [1]. As a benign disease, the pathogenesis of CD remains unclear, and there is still a lack of effective treatment. Further research is needed to explore more effective treatments for CD. Previous studies suggested that chronic wall-penetrating inflammation commonly seen in the bowel of patients with CD was mediated by T lymphocytes. Migration away or in-situ loss of dendritic cells (DCs) from the terminal ileum might be responsible for the significant reduction of DCs in CD patients [2]. Liu et al. carried out an in silico analysis of immune cell populations in colon samples from IBD patients and found significant enrichment of mononuclear phagocytes including DCs in inflammatory tissue, which needs to be carefully teased out for their role in the pathogenesis of IBD [3].

5-HT7 receptor (5-HT7R), a member of the 5-HT receptor family that recently received extensive attention, with most of the current relevant studies focusing on its role in neural development [4]. A recent study suggested that 5-HT released by intestinal enterochromaffin (EC) cells, which works by binding to 5-HT7R in DCs, may increase inflammation [5]. The 5-HT7R-mediated signal played an important role in the regulation of DCs morphology and movement. This suggested that 5-HT7R may be a new target for the treatment of various inflammatory and immune diseases [6]. Yu et al. Studied the difference in visceral sensitivity of colonic mucosa between patients with diarrhea type irritable bowel syndrome (IBS-D) and patients with remission IBD, and linked these differences with changes in 5-hydroxytryptophan (5-HT) signaling pathway. It was found that IBS-D group and IBD group showed similar changes in clinical symptom scores, visceral sensitivity and serotonin signaling pathway indexes in plasma and colon mucosa. However, the expression of 5-HT7R was significantly increased in the colonic mucosa of IBD patients [7]. At the same time, several recent studies have shown that drug intervention targeting 5-HT7R may be a potential therapeutic strategy to improve mucosal inflammation and intervene in inflammatory diseases (such as IBD). More studies are needed to further understand the therapeutic potential of 5-HT7R in inflammatory bowel diseases [8, 9].

To our best of knowledge, the effects of 5-HT7R on DCs regulation were all demonstrated with animal models, therefore the research conclusions were controversial [10]. The current study intends to further explore the influence of 5-HT7R on persistent autoimmune inflammation in CD patients in clinical specimens in order to find novel therapeutic targets for CD mucosal inflammation.

## Materials and methods

### Study design and patients

Patients suspected with CD admitted to the Department of Gastroenterology, the First Affiliated Hospital of Anhui Medical University from June 2014 to January 2018 were enrolled in this study. A total of CD 112 patients were diagnosed according to the Asia Pacific consensus statements on CD diagnosis and treatment [11], among which 50 patients were enrolled in the present study including 28 in the active stage and 32 in the remission stage after screening based on the following criteria. The inclusion criteria are (1) patients with clinically diagnosed Crohn’s disease who meet the diagnostic criteria of Asia Pacific consensus statements on Crohn’s disease [11], (2) patients with complete basic information, medical history, serological examination, endoscopy and imaging examination data, and (3) patients whose interval between the above examinations is not more than 1 week. The exclusion criteria include (1) those with contraindications of colonoscopy or magnetic resonance examination, (2) there are more than two sequences of MRI with poor image quality, (3) the total amount of oral contrast agent is less than 1000 ml, and (4) poor bowel clearing effect affects the observer of enteroscopy.

After the matching of gender and age, another 20 patients with no obvious abnormalities who underwent colonoscopy in our hospital during the same period were enrolled as controls. Information of gender, age, course of disease, smoking history, surgical history, clinical symptoms, laboratory tests, colonoscopy, endoscopic CD scores (SES-CD), magnetic resonance imaging results and MaRIA scores were recorded from the all participants. Base on the Best CDAI score [12], the patients were divided into control group, remission group and active group. All clinical data were verified and presented in Table 1. The study with all the procedures were approved by the Biomedical Ethics Committee of Anhui Medical University (No. 20,190,098).

**Table 1:** Demographic data and diagnostic test results of the 50 enrolled patients

Characteristics	control group	remission group	active group
Age (Years), Mean ± SD	31.15 ± 5.89	27.09 ± 6.59	27.54 ± 6.44
Gender, n (%)			
Male	9(45.0%)	17(53.1%)	15(53.6%)
Female	11(55.0%)	15(46.9%)	13(46.4%)
Course (Years), Mean ± SD		3.65 ± 2.45	3.96 ± 2.44
Location			
Ileum, n (%)		2(6.2%)	1(3.6%)
Ileocolon, n (%)		18(56.3%)	22(78.6%)
Colon, n(%)		12(37.5%)	5(17.8%)
Classification			
Penetrating, n(%)		5(15.6%)	7(25.0%)
Stricturing, n(%)		21(65.6%)	16(57.2%)
Non-stricturing & non-penetrating, n(%)		6(18.8%)	5(17.8%)
Perianally lesions, n(%)		3(9.4%)	6(21.4%)
Operation history, n(%)		5(16.6%)	9(32.1%)
Smoking history, n(%)	2(10.0%)	4(12.5%)	6(21.4%)
Best CDAI Score, Mean ± SD	19.35 ± 10.97	102.09 ± 28.79	329.83 ± 108.69
SES-CD Score, Mean ± SD	1.45 ± 0.94	15.07 ± 3.26	6.43 ± 1.68
MaRIA Score, Mean ± SD	3.51 ± 1.86	12.14 ± 1.22	49.77 ± 13.46
Laboratory tests			
ESR (mm/h) ,Mean ± SD	5.60 ± 2.11	15.01 ± 4.80	36.50 ± 8.22
CRP (mg/L), Mean ± SD	6.25 ± 2.24	23.03 ± 4.76	65.85 ± 14.12
WBC (×109/L), Mean ± SD	6.43 ± 1.28	8.72 ± 1.21	13.95 ± 1.65
HB (g/L), Mean ± SD	132.95 ± 9.13	95.73 ± 9.58	70.60 ± 9.46
HCT (%), Mean ± SD	40.17 ± 2.97	34.17 ± 2.84	29.29 ± 1.95
ALB (g/L), Mean ± SD	47.77 ± 4.64	35.71 ± 2.81	28.45 ± 2.12

### Laboratory tests

Erythrocyte sedimentation rate (ESR) and C-reactive protein (CRP) [13] have long been considered as inflammatory markers associated with CD activity, with high diagnostic sensitivity but insufficient specificity. CRP is one of the most studied indicators of inflammation. Previous studies suggested that increased CRP was positively correlated with CD activity, which increased significantly in the early stage of inflammation and decreased rapidly after treatment. Thus, CRP will be biased in its assessment of CD activity when CD patients treated with medication before laboratory tests. Compared to CRP, ESR rose and fell more slowly during the disease. Leukocytosis was also common in active CD but with low specificity. The number of platelets might increase in the inflammatory state but the range of normal values was too wide and the sensitivity and specificity were poor. Decreased serum albumin (ALB) levels may be observed in active CD patients with malnutrition, malabsorption, and loss of intestinal proteins. Hemoglobin (Hb), width distribution of erythrocyte volume, D-dimer, and fecal calprotectin have also been considered as other indicators of CD activity.

### Colonoscopy

All patients were instructed to intake easily digestible foods the day before the examination and fast for eight hours prior to the examination. After the polyethylene glycol was diluted to 3000 mL of warm water, the solution was taken orally 4-6 h before the examination until the patient’s discharge was clear. Before colonoscopy, digital rectal examination was performed to determine whether there was anal fissure, internal hemorrhoids, rectal stricture and other abnormal conditions. The patient was examined in left lateral decubitus position with legs flexed. Colonoscopy procedure was performed by an endoscopist with at least five years of experience in colonoscopy. During the procedure, multiple bowel segments were biopsied at the terminal ileum, ileocecal region, ascending colon, descending colon and sigmoid colon respectively. Two biopsy tissue pieces were collected sequentially at the same location in the bowel, each is about 5 × 5 mm in size. The tissue was placed respectively in Trizol reagent for RNA extraction and in 4% fixative for immunofluorescence. Endoscopists were blinded to data such as magnetic resonance or laboratory tests. The severity and extent of endoscopic lesions were assessed using the simplified Crohn’s disease endoscopic score (SES-CD) [14].

### Magnetic resonance imaging

All patients were diagnosed by magnetic resonance enterography using SIEMENS Magnetom Avanto1.5T MRI scanner and body coils. Patients were required to ingest liquid food 24 h before the examination, and clean the enema if necessary. Forty-five minutes before the examination, 500 ml x 3 of 2.5% mannitol were taken orally with an interval of 15 min. Anisodamine at 20 mg was injected intravenously before the examination. Imaging sequence parameters for small intestine were set as FOV = 400 ~ 550 mm, layer thickness 3.5 ~ 6 mm, interval 1.7 ~ 4.2 mm, and breath-hold scan with rapid sequence. Normal scan and contrast-enhanced scan were carried out separately. MaRIA score was applied to each patient. The calculation formula was MaRIA score = 1.5 × intestinal wall thickness (mm) + 0.02 × RCE + 5 × edema + 10 × ulcer. In the current study, the ileocolon was divided into five segments: terminal ileum, ileocecal portion, ascending colon, descending colon and sigmoid colon. The thickest intestinal wall of each intestinal segment was recorded as the intestinal wall thickness in the MaRIA scoring formula. Mucosal ulcer was defined as a deep depression of the thickened intestinal wall. Intestinal wall edema was defined as a strong T2-weighted sequence compared to the psoas major signal intensity. Edema or ulceration in the intestinal segment was scored 1 point, or 0 points if none exists. Related contrast enhancement (RCE) was measured in the areas with the maximum signal strength in each segment. The MaRIA score was global MaRIA which was the sum of the MaRIA scores of each intestinal segment. A MaRIA score of each intestinal segment ≥ 7 was the indication of disease activity and ≥ 11 represented severe activity [15].

### Immunofluorescence

Each patient was sampled from at least five sites including terminal ileum, ileocecal, ascending, descending, and sigmoid colon. Biopsy samples were immobilized in freshly prepared 4% PFA and histological and immunofluorescence analyses were performed. The slices with frozen sections of biopsy tissue were washed with PBS and incubated for one hour at room temperature with 0.2% Triton X-100 (Sigma-Aldrich), 0.02% sodium azide (Merck), and 5% normal donkey serum (Jackson ImmunoResearch Laboratories). Next, the slices were incubated with primary antibody (rabbit anti-5-HT7R Antibody, BS-12056R, ThermoFisher Scientific; mouse anti-CD11c Antibody, BDB-550,375, BD Biosciences; or goat anti-CD86 Antibody SC-28,347, Santa Cruz Biotechnology) diluted with 0.5% carrageenan (Sigma-Aldrich) and 0.02% sodium azide in PBS for 3 days at 4℃. After washing with PBS, the slices were placed in PBS/carrageenan solution at room temperature and incubated with 1:200 ~ 1:400 diluted secondary antibodies (Alexa 594 conjugated anti-rabbit IgG from Life Technologies, Carlsbad, CA, FITC-conjugated anti-mouse IgG from Biolegend, Fell, Germany, or DyLight 405 conjugated anti-goat IgG from Jackson ImmunoResearch Europe) for 2 h. The slices were sealed with 80% propylene glycol and the fluorescence distribution was observed under a fluorescence microscope. The fluorescence intensity of the samples was analyzed by Media Cybernetic Express (USA).

### Semi-quantitative PCR

**Fig. 1:**
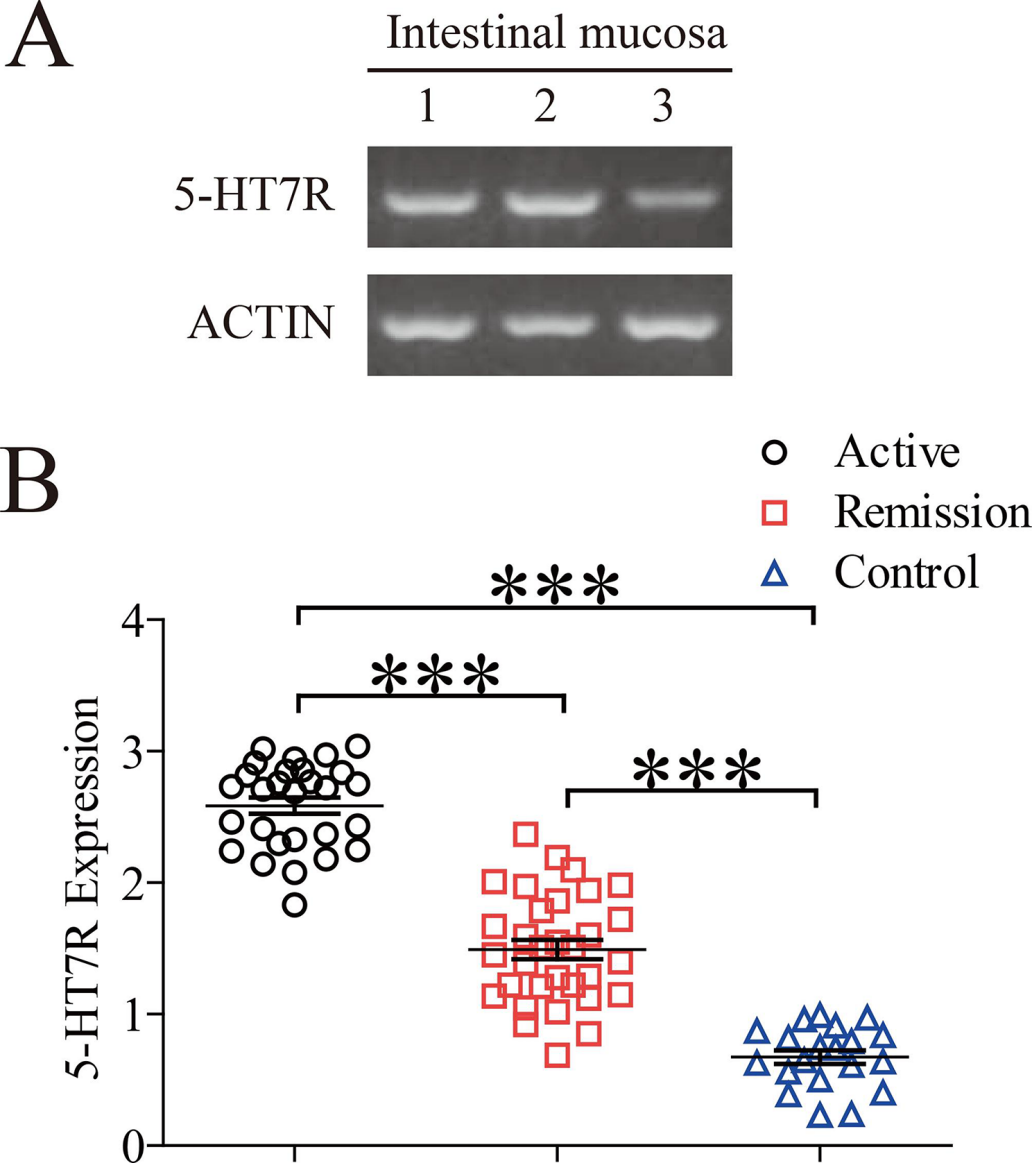
Expression of 5-HT7R mRNA in the inflammatory region of intestinal mucosa ((A) Expression of 5-HT7R mRNA and β-actine mRNA in the remission group (Lane 1), the CD active group (Lane 2) and control group (Lane 3) respectively. (B) Scatter plots of 5-HT7R mRNA expression in intestinal mucosa in the patients of the three groups. Data represent the mean ± SEM. * p < 0.05; ** p < 0.01; *** p < 0.01.)

Patient samples were frozen with liquid nitrogen immediately after collection. Total RNA was extracted and cDNA was synthesized with PrimeScript 1st strand cDNA synthesis kit from Takara Bio USA. The expression of β-actin was analyzed with the Human ACTB Endogenous Reference Gene Kit (Sangon Biotech, Shanghai, China). The upstream primer of 5-HT7R was 5′-GCTCATCACGCTGACGAT-3′, and the downstream primer was 5′-CGCCAGGGACACAATCAGG-3′. The amplicon size was 106 bp, and the experimental procedure was performed according to the kit instructions. For the electrophoresis analysis of PCR products, 8 µL PCR products were loaded in 2% agarose gel for electrophoresis. The resulting gel image was recorded and analyzed by StepOne Software v2.1 (Applied Biosystems, Darmstadt, HE, Germany). The numbers 0 to 4 of the Y-axis in Fig. 1B represent the mRNA expression levels by the semi-quantitative PCR.

### Statistical analysis

SPSS 22.0 software was used for statistical analysis. Quantitative data were expressed as mean of standard deviation (mean ± SD), and counting data were expressed as counts and occurrence frequency (%). Independent sample T test was used for the mean comparison between the two groups. The mean comparison among the three groups was performed by one-way ANOVA and corrected by Bonferroni method. The correlations between 5-HT7R expression and MaRIA, SES-CD and clinical disease activity evaluation indexes were analyzed by Spearman rank coefficient, and the scatter plot was generated by SPSS software. The measurement data were compared by Bonferroni method. The Chi-square test was used for counting data analysis.

## Results

### Expression and distribution of 5-HT7R in intestinal mucosal tissues by immunofluorescence

**Fig. 2:**
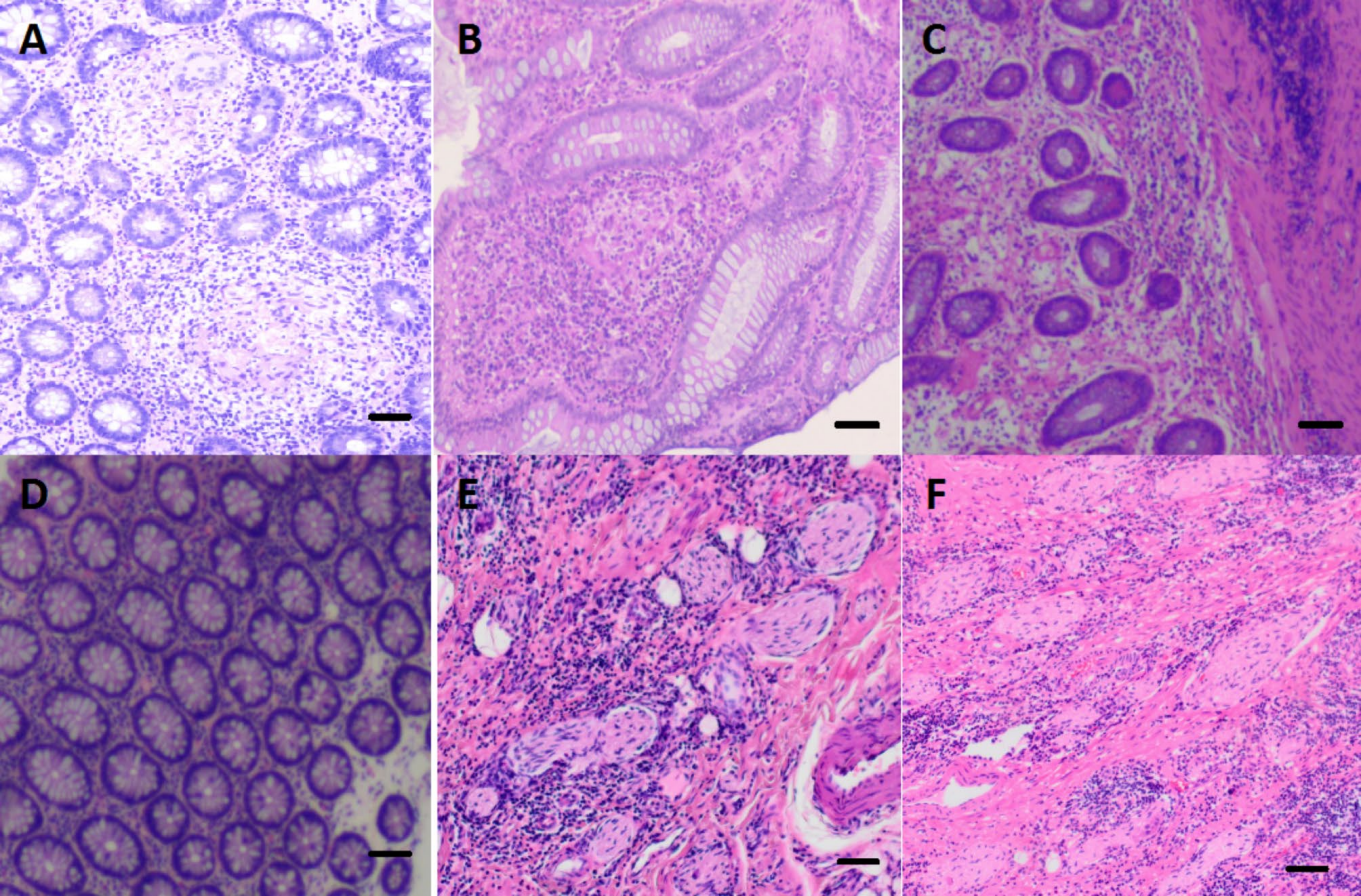
Intestinal mucosal histology and pathology (Histological images of intestinal mucosa in the active group (A & B), remission group (C) and control group (D). Distribution of inflammation in the intestinal plexus in the active group (E). Distribution of inflammation in the intestinal plexus in the remission group (F). Scale bars were 50 μm.)

**Fig. 3:**
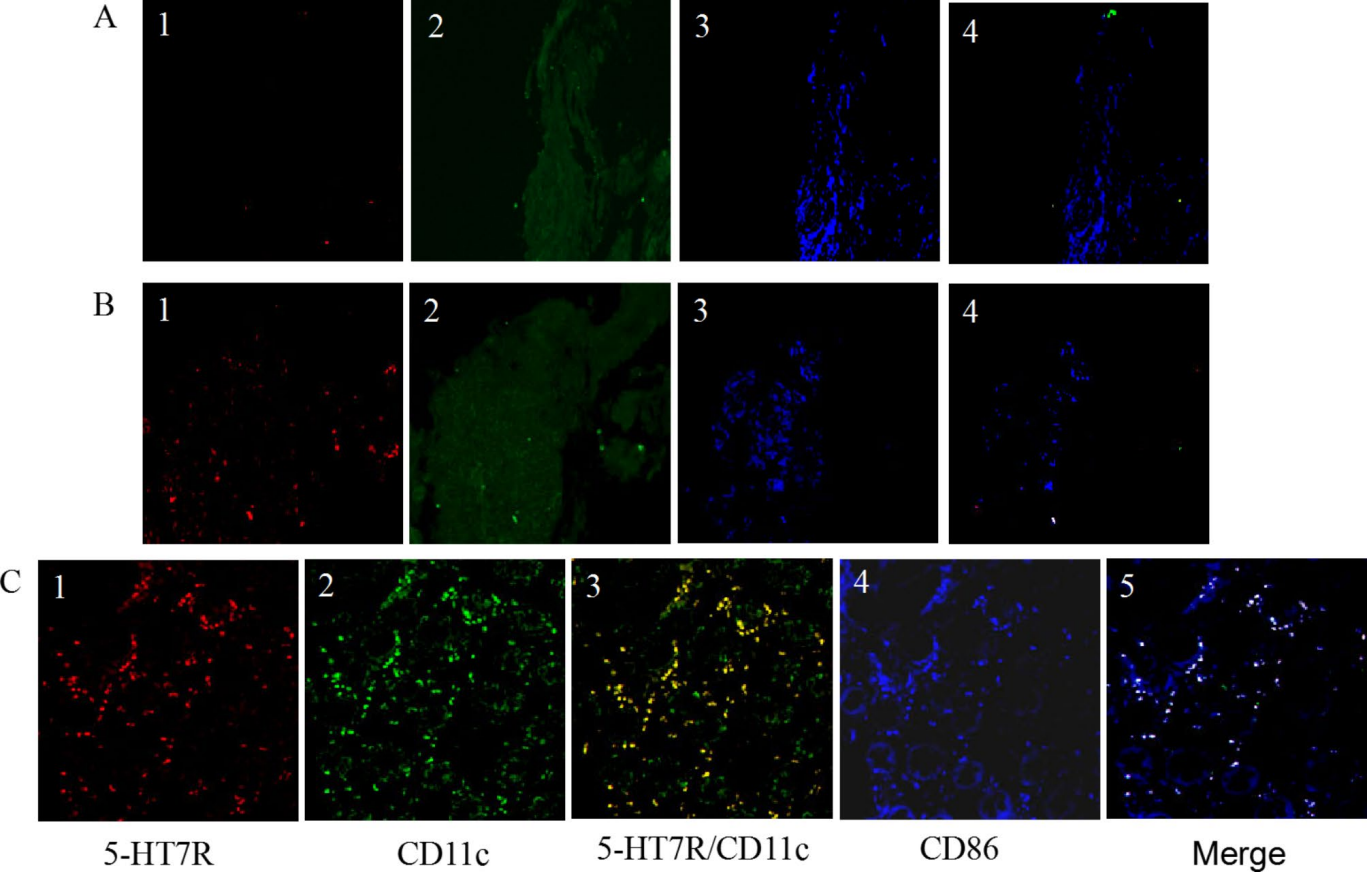
Expression of 5-HT7R in inflammatory regions of intestinal mucosa by immunofluorescence ((A) Expression of 5-HT7R (A1), CD11c (A2), and CD86 (A3) in the control group. A1, A2 and A3 were merged to show co-localization of 5-HT7R/CD11c/CD86 in the control group (A4). (B) Expression of 5-HT7R (B1), CD11c (B2), and CD86 (B3) in the remission group. The co-localization of 5-HT7R/CD11c/CD86 in the remission group was shown in B4. (C) Expression of 5-HT7R (C1), CD11c (C2) and CD86 (C4) in the active CD group. The co-localization of 5-HT7R/CD11c (C3) and 5-HT7R/CD11c/CD86 (C5) were shown respectively in the active CD group.)

Firstly, the intestinal mucosa among the three groups was histologically analyzed. Compared with those in the remission group and the control group, the mucosa tissue from patients in active CD group showed extensive inflammatory infiltration and marked edema in the intestinal mucosa and muscle adventitia (Fig. 2A-D). Interestingly, caseous granulomas (Fig. 2A) and damaged epithelium in intestinal crypts (Fig. 2B) were frequently observed in the intestinal mucosa during active disease. An experienced pathologist was invited to observe the distribution of inflammation in the intestinal submucosal plexus or intermuscular plexus (Fig. 2E-F). Secondly, Immunofluorescence showed no expression of 5-HT7R in the normal control group. Compared with that in the remission group, the expression of 5-HT7R in the active CD group was significantly increased (Fig. 3A). Immunofluorescence analysis of intestinal biopsy specimens from the three groups demonstrated that the expression level of 5-HT7R was significantly increased in the regions with significant intestinal inflammation (Fig. 3A, 3B & 3C). Most of the intestinal mucosal cells expressing 5-HT7R in the inflammatory region were positive for CD11c (Fig. 3C2). We quantitatively analyzed the colocalization of CD11c and 5-HT7R by calculating the overlap coefficient, and found that the colocalization between CD11c positive cells and 5-HT7R spots was 88.5% ± 2.16% (Fig. 3C3) in the inflammatory area of the intestinal mucosa in the CD active group. In addition, CD86 expression was positive in these cells (Fig. 3C4), and the colocalization of 5-HT7R/CD11c/CD86 was 85.2% ± 1.41% (Fig. 3C5) in the tissue from the same group.

**Fig. 4:**
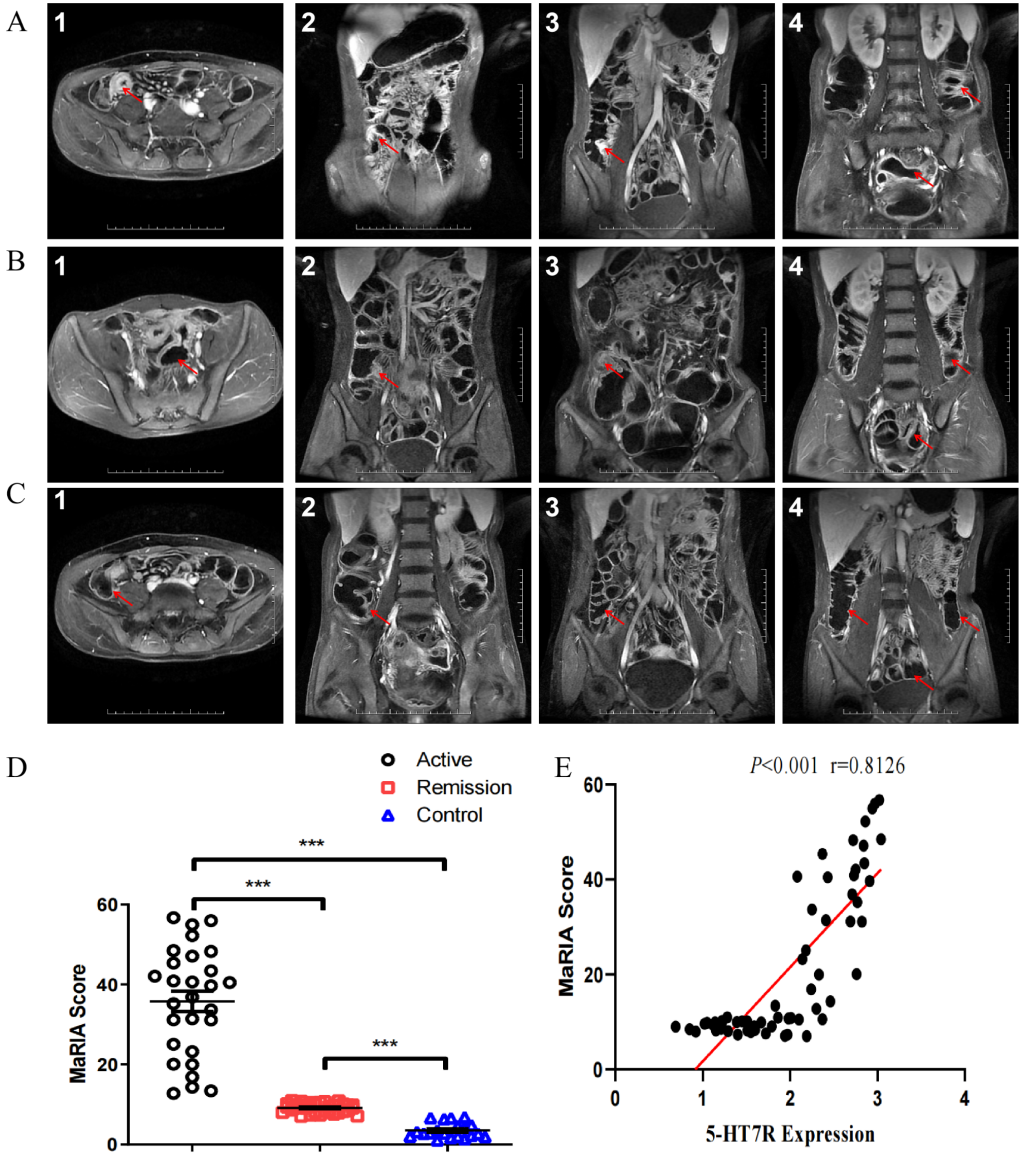
Magnetic resonance images of inflammatory regions of the intestine (T1WI enhanced images of terminal ileum in transverse axis (1), terminal ileum (2), ileocecal junction (3), descending colon, and sigmoid colon (4) in the CD active group (A), the remission group (B) and the control group (C). (D) Scatter plot of MaRIA scores. (E) Linear correlation analysis between MaRIA score and 5-HT7R expression in CD patients.)

**Fig. 5:**
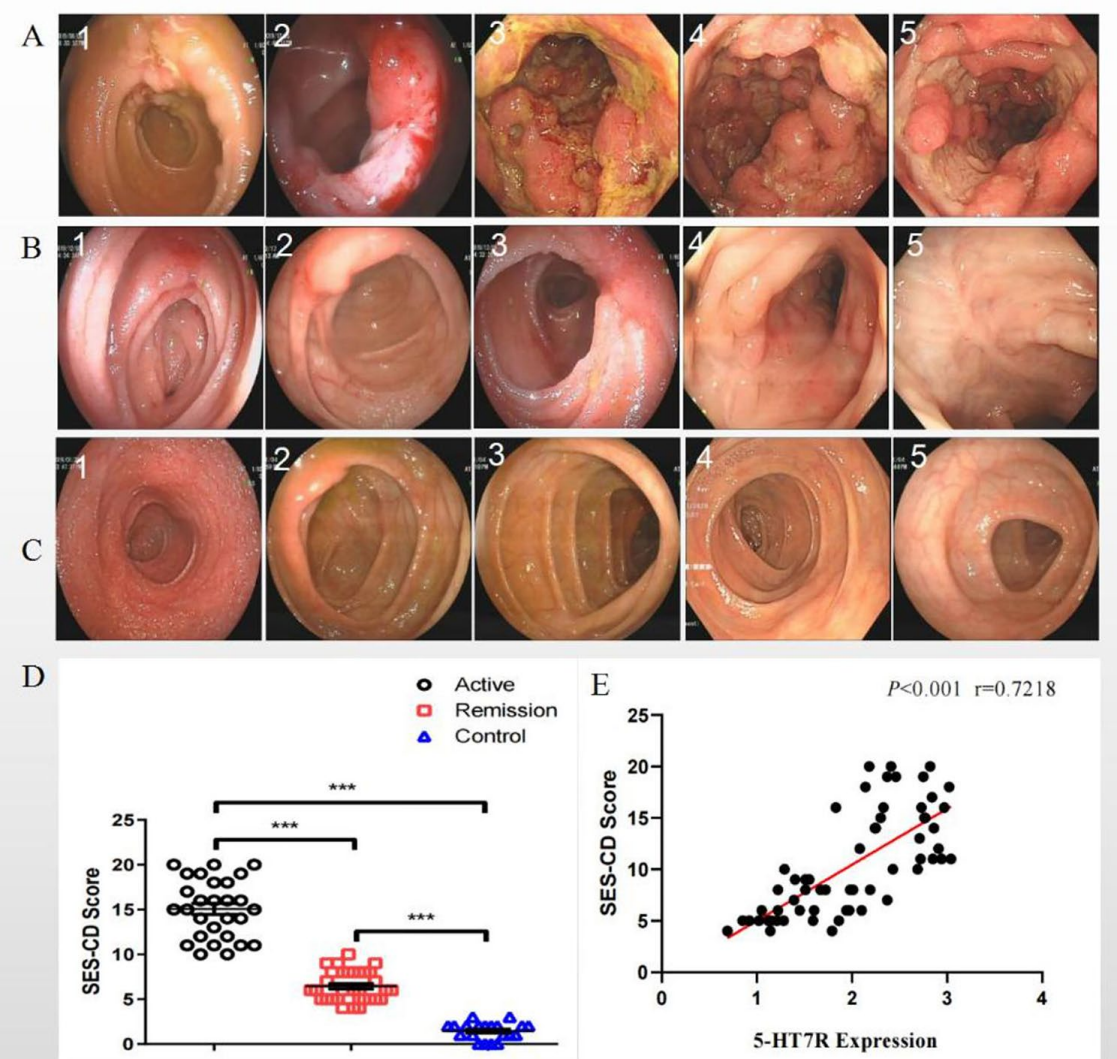
Typical endoscopic images and SES-CD score(Typical endoscopic images of terminal ileum (1), ileocecal junction (2), ascending colon (3), descending colon (4), and sigmoid colon (5) in the CD active group (A), the remission group (B) and the control group (C). (D) Scatter plot of SES-CD scores. (E) Linear correlation analysis between SES-CD score and 5-HT7R expression in CD patients.)

### Expression of 5-HT7R mRNA in intestinal mucosa

The mRNA expression of 5-HT7R in the three groups was analyzed by semi- quantitative PCR. 5-HT7R expression was found in all samples of the three groups. The expression level of 5-HT7R was the lowest in the control group, and the highest in the active CD group. The mRNA expression level of 5-HT7R in the active group was significantly higher than that in the remission group (Fig. 1A), and was about 3 times higher than that in the control group (Fig. 1B).

Based on the results shown in Figs. 3 and 1, it might suggest that the increased expression of 5-HT7R promoted the recruitment and aggregation of DC cells that may mediate the development of intestinal mucosal inflammation.

### The correlation between 5-HT7R expression and magnetic resonance imaging

Magnetic resonance images of typical CD cases were shown in Fig. 4. The expression of 5-HT7R was closely correlated with magnetic resonance images, including intestinal wall thickness, enhanced intestinal wall signal intensity, relevant contrast enhancement (RCE), edema, and ulcer among others. Particularly, the mean of intestinal wall thickness in the active group was 7.61 mm, which was significantly higher than that of the remission group (3.26 mm) and the normal control group (2.53 mm). Similarly, the mean of intestinal wall thickness in the remission group was also higher than that of the normal control group (Fig. 4A, 4B & 4C). There was no significant difference in the signal intensity among the three groups. After MRI contrast agent enhancement, the signal intensity in the CD active group (462.32) was significantly higher than that in the remission group (361.71) and the control group (302.43). The signal intensity of remission group was also significantly higher than that of control group.

RCE expression in the CD active group (146.22) was significantly higher than that in the remission group (98.71) and the control group (82.15). Edema was found in 36% of the intestine of the CD active group, significantly higher than that of the remission group (7%) and the control group (0%). There was no statistically significant difference in the incidence of edema between the remission group and the control group.

Intestinal ulcer was found in 8.2% of the CD active group, significantly higher than the remission group (0%) and the control group (0%). The MaRIA score of the active group was significantly higher than that of the remission group and the control group (Fig. 4D). The correlation between 5-HT7R expression and total MaRIA score in CD patients was analyzed. The linear correlation coefficient r = 0.8126, P < 0.001 (Fig. 4E).

### The correlation between endoscopic manifestations, SES-CD score and 5-HT7R expression

Typical endoscopic images of the CD active group, the remission group and the control group were shown in Fig. 5A, 5B and 5C, respectively. SES-CD scores were calculated based on parameters including ulcer area, ulcer size and lesion range as shown in the endoscopic images. The SES-CD score of the active group was significantly higher than that of the remission group and the normal control group, and the MaRIA score of the remission group was higher than that of the control group (Fig. 5D). CD disease was considered to be active when SES-CD score ≥ 1 [11], and the linear correlation between 5-HT7R expression and endoscopic lesion severity was analyzed. In CD patients, the linear correlation coefficient, r, of 5-HT7R expression and corresponding SES-CD scores in the terminal ileum, ileocecal, ascending colon, descending colon, sigmoid colon and all intestinal segments were 0.73, 0.79, 0.75, 0.76, 0.77, and 0.72, respectively. These data support a significant correlation between 5-HT7R expression and SES-CD score (Fig. 5E).

### Correlation of other parameters with the expression of 5-HT7R

**Fig. 6:**
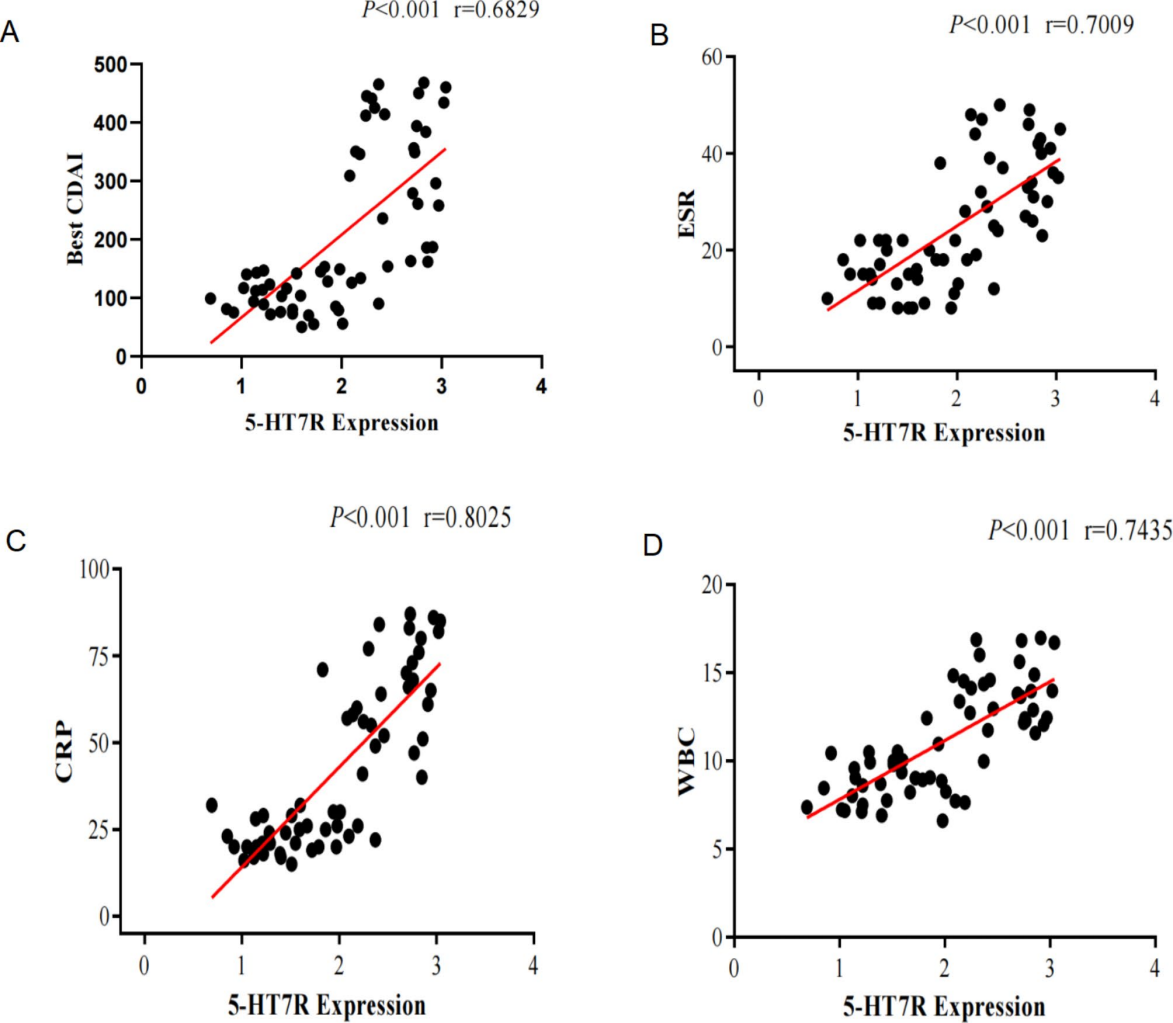
Correlation of other parameters with 5-HT7R expression (Correlation analysis between 5-HT7R expression and best-CDAI (A), ESR (B), CRP (C) and WBC (D) in CD patients respectively.)

We then analyze the correlation between 5-HT7R expression in the gut with other clinical symptomatic indicators (Best-CDAI) as well as with laboratory blood tests (CRP, WBC, ESR) in CD patients. Correlation coefficient r between 5-HT7R expression and Best-CDAI, ESR, CRP, WBC were 0.6829 (Fig. 6A), 0.7009 (Fig. 6B), 0.8025 (Fig. 6C) and 0.7435 (Fig. 6D), respectively. Spearman’s rank correlation coefficient analysis showed that 5-HT7R expression was positively correlated with Best-CDAI, CRP, ESR and WBC.

## Discussion

CD is a lifelong disease with unknown etiology, characterized by a chronic alteration of relapse-remission. Current medications may relieve clinical symptoms and control intestinal inflammation, but is not able to cure CD [16]. Patients with CD in the active phase usually required either a single steroid administration or a combination therapy with immunosuppressive agents, or even biologic therapy to induce remission. Non-steroidal immunosuppressants were often required to maintain remission. Recent studies, however, demonstrated that some drugs used to treat CD and gut inflammation, such as steroid hormones, might increase the risk of heart disease including coronary heart disease and heart failure, especially in women and young adults at increased risk for acute myocardial infarction and stroke [17]. Surgical intervention would be considered when clinical symptoms were difficult to control by medications available. Complications such as perianal disease, stenosis and intraperitoneal fistula will increase the surgical risk of CD patients. Furthermore, the treatment of these complications was difficult, which requires close cooperation between gastrointestinal surgeons and gastroenterologists [18]. All of those contributed to high morbidity and mortality of CD. Therefore, early diagnosis, appropriate treatment and control of inflammation were of great importance to improve the quality of life and prognosis of patients.

Enteric serotonin (5-HT) not only plays vital roles as a growth factor, a hormone, a paracrine factor and a neurotransmitter in gut, but also is an important mediator involved in a range of gastrointestinal inflammatory diseases. Enterochromaffin (EC) cells secrete 5-HT.and those cells are located throughout the intestinal tract from the stomach to the colon [19]. Alterations in the number of EC cells and in the quantity of 5-HT that EC cells produced could be observed in both IBD patients and animal models of colitis. It was reported that increased mucosal 5-HT signaling or increased 5-HT utilization may contribute to the inflammation in CD patients [20].

5-HT perform its biological functions by activating specific receptors, which consist of 7 different types based on structural and functional characteristics. Five members of the receptor family (5-HT1R, 5-HT2R, 5-HT3R, 5-HT4R and 5-HT7R) were expressed in intestinal neurons and participated in the regulation of gastrointestinal movement [21]. Initially 5-HT7R was considered to be able to regulate the motility of ileum, stomach and colon by regulating smooth muscle activity. Therefore, the biological function of 5-HT7R in IBD was related to the number of receptors expressed on smooth muscle and intestinal neurons, and participated in the regulation of the circular muscle adaptation in the early stage of ileum periperiasis, leading to abdominal distension. Recent studies also suggested that 5-HT7R might play an important role in the pathogenesis of IBD inflammation [22].

Previous studies in animal models found that 5-HT functions in intestinal inflammation by binding to 5-HT7R on the surface of DCs [23]. An additional observation by Guseva et al. [24] demonstrated that in gut 5-HT7R expressed by the intestinal CD11c/CD86 double-positive cell subsets, which suggest those CD11c^+^/CD86^+^ cells may play a key role in the onset and the development of intestinal inflammation. Pharmacological blockage or genetic deletion of 5-HT7R was able to aggravate intestinal inflammation; in contrast, activation of 5-HT7Rs promoted anti-inflammatory responses.

Furthermore, it was found 5-HT7R expression in DCs was significantly up-regulated in the lamina propria in inflammatory mouse model [21]. The current study demonstrated that the 5-HT7R positive cells were mainly located in both the neuronal intermuscular plexus and submucosal plexus, especially in the annular muscularis and intermuscular plexus (Fig. 3). This is consistent with the data shown in animal studies by Prause et al. [25]. Our histological analysis showed that CD patients presented with inflammatory infiltration of colonic mucosa and musculoskeletal, epithelial injury and crypt shape disorder. When 5-HT7R expression was significantly elevated, pathological damages in the same tissue were more significant. In contrast, there was little change in the morphology of the intestinal wall in the control group (Fig. 4). The biological function of 5-HT7R in IBD was similar to the mechanism of a series of symptoms caused by ileum peristalsis in functional bowel disease [7]. The role of 5-HT7R in IBD was thought to be mainly related to smooth muscle and receptors expressed on enteric neurons, as well as to the adaptation of circular muscles in the early stage of ileal peristalsis, which may lead to some similar symptoms to functional intestines disease.

In the current study, intestinal biopsies and blood samples from the same CD patients were used to analyze the role of 5-HT7R in CD. The results showed that 5-HT7R expressed by the intestinal CD11c^+^/CD86^+^ cell subsets is likely played a key role in the onset and clinical process of intestinal inflammation. Since the activation of 5HT7R may promote the anti-inflammatory response in the gut, expression of 5-HT7R could aggravate enteritis in patients with CD. The increased expression of 5-HT7R in the intestinal immune cells of CD patients further suggested the role of 5-HT7R in inflammation. Therefore, activation of 5-HT7R signaling pathway might play an important role in the progression of inflammation.

It is interesting that 5-HT7R expression was significantly increased in the inflammatory regions of the intestinal tract in CD patients. 5-HT7R was mainly expressed on CD11c^+^/CD86^+^ cells, which suggests the important role of 5-HT7R in regulating the function of mature DCs in the mucosal immune response of patients with CD. In addition, the expression of 5-HT7R was positively correlated with CDAI, SESCD, and MaRIA scores, suggesting that 5-HT7R expression was consistent not only with the clinical manifestations of intestinal inflammation, but also with the mucosal inflammation of the intestinal wall and the alterations of inflammatory cells infiltrating the intestinal wall including mucosa, submucosa, and serosa.

Accurate determination of CD disease severity is imperative in personalized medication [26]. Evaluation of CD disease severity includes clinical severity evaluation, intestinal mucosal injury evaluation and intestinal penetrative inflammatory exudate evaluation. CDAI, SES-CD, and MaRIA scores were commonly used to assess clinical severity, endoscopic intestinal mucosal injury, and intestinal transmural inflammatory exudation, respectively. SES-CD score is used to judge CD activity directly and objectively. Pathology observations suggested that multiple inflammatory cell infiltration and non-caseous granuloma formation were of great significance for the diagnosis of CD [27]. Penetrative inflammatory exudation and parenteral complications might be identified by MaRIA. Changes in the severity of the lesions caused by treatment can be accurately detected by thickening of the intestinal wall, enhancement of relevant contrast, tooth comb or mesangial vessel dilation and tortuosity. Therefore, MaRIA may be used to evaluate mucosal healing [28]. According to numerous clinical studies, most of the laboratory indicators used to determine the activity of clinical diseases, such as ESR and CRP, were inconsistent with the extent of intestinal mucosal injury and MRI results. And there was no significant correlation between these indicators and SES-CD and MaRIA scores. Therefore, these indicators cannot truly reflect the severity of inflammation in the intestinal mucosa [29]. However, the CDAI score was based on the subjective feeling of the patients as the gold standard, and the symptoms and signs of abdominal pain, diarrhea, abdominal mass and complications of CD patients were scored, without the mucosal inflammation being included in the scoring range [30]. Thus, it is important for clinicians to find a laboratory indicator that can reflect the severity of CD disease.

Our study showed that 5-HT7R was differentially expressed between the CD active group and the remission group, indicating its significance in differentiating disease activity status. In addition, 5-HT7R expression was well correlated with MaRIA, SESCD, CDAI scores and inflammatory markers (WBC, CRP & ESR). Although it was reported that CRP, CDAI and WBC had a low correlation with SES-CD score (0.3 < r ≤ 0.5) [29, 30], our study showed that 5-HT7R expression was strongly correlated with SES-CD (r > 0.8). Endoscopy is an invasive procedure that leaves a painful experience to patients, and in addition the procedure has some risks of perforation, bleeding, and unsuccessful examination. In addition, 5-HT7R expression was also correlated with MaRIA scores (r = 0.8126, P < 0.001).

MRI played an irreplaceable role in the evaluation of CD condition. For example, thickening of the intestinal wall and high signal intensity of T2 were considered as effective markers of CD activity, but its disadvantages such as high price and high consumption of liquid before examination limited its wide application. Serological markers such as WBC, CRP and ESR may represent systemic inflammation status but tthey are lack of intestinal specificity, while CDAI scores were not reliable indicators for disease and severity. Therefore, 5-HT7R expression in gut could be a potentially non-invasive, convenient, economic and cross-check approach to evaluate inflammatory activity in CD patients.

In summary, the present study found that 5-HT7R expression was significantly increased in the inflammatory regions of human intestine in CD patients. 5-HT7R was mainly expressed by CD11c/CD86 double positive cells, indicating that 5-HT7R might play an important role in regulating the function of mature DCs in mucosal immune response. Additionally, 5-HT7R expression may be used as an indicator to evaluate CD intestinal inflammation in clinical practice, so as to accurately determine disease stage, inflammatory activity and prognosis, and then implement individualized treatment. Overall, our data suggested that 5-HT7R might serve as a novel therapeutic and evaluation target for CD mucosal inflammation.

The clinical manifestations of CD are complex and it usually is not straightforward to make diagnosis. At present, there are no reliable and clinically applicable biomarkers. We now provided evidence that the gene expression of 5-HT7R is correlated with the clinical, radiological, and endoscopic activity scores of CD patients, which suggests that 5-HT7R expression might be an applicable biomarker to evaluate the disease severity of CD. However, more research is needed to evaluate the 5-HT7R gene expression with the pathological features in the affected area of the bowel of the patients with CD, as well as with the status of disease recurrence and drug efficacy in patients with CD. Additional prospective studies with standardized experimental procedure could be carried out to establish a cut-off value to facilitate clinical application.

## Data Availability

The datasets generated during and/or analyzed during the current study are available from the corresponding author on reasonable request. We do not wish to share data because it is identifying or confidential patient data.

## Abbreviations

CD: Crohn’s disease.
IBD: Inflammatory bowel disease.
5-HT: 5-hydroxytryptamine.
5 -HT7R: 5-hydroxytryptamine 7 receptor.
CDAI: Clinical disease activity.
SES-CD: CD endoscopic score.
MaRIA: Magnetic resonance score.
DCs: Dendritic cells.
EC: Pheochromocytoma.
ESR: Erythrocyte sedimentation rate.
CRP: C-reactive protein.
ALB: Albumin.
Hb: Hemoglobin.
RCE: Related contrast enhancement.

## References

[1] Buttó LF, Schaubeck M, Haller (2015). Mechanisms of microbehost interaction in Crohn’s disease: dysbiosis vs. pathobiont selection. Front Immunol.

[2] Faleiro R, Liu J, Karunarathne D (2019). Edmundson ACrohn’s disease is facilitated by a disturbance of programmed death-1 ligand 2 on blood dendritic cells. Clin Transl Immunology.

[3] Liu H, Dasgupta S, Fu Y, Bailey B, et al. Subsets of mononuclear phagocytes are enriched in the inflamed colons of patients with IBD. BMC Immunol,2019; 12;20:42. 10.1186/s12865-019-0322-z PMC6852755 31718550

[4] De Deurwaerdère P, Bharatiya R, Chagraoui A (2020). Constitutive activity of 5-HT receptors: Factual analysis. Neuropharmacology.

[5] Janice JK, Byram WB, Jean-Eric G (2013). Targeted inhibition of serotonin type 7 (5- HT7) receptor function modulates immune responses and reduces the severity of intestinal inflammation. J Immunol.

[6] Holst K, Guseva D, Schindler S (2015). The serotonin receptor 5-HT_7_R regulates the morphology and migratory properties of dendritic cells. J Cell Sci.

[7] Huang Feng-YanYShao-Gang, Zhang H-Y (2016). Comparison of 5-hydroxytryptophan signaling pathway characteristics in diarrhea-predominant irritable bowel syndrome and ulcerative colitis. World J Gastroenterol.

[8] Kevin M, Blattner DJ, Canney, Douglas A, Pippin (2019). Pharmacology and Therapeutic Potential of the 5-HT 7 Receptor. ACS Chem Neurosci.

[9] Alejandro Quintero-Villegas. Sergio Iván Valdés-Ferrer.Role of 5-HT 7 receptors in the immune system in health and disease.Mol Med. 2019 31; 26(1):2. 10.1186/s10020-019-0126-x PMC6938607 31892309

[10] Guseva D, Holst K, Kaune B (2014). Serotonin 5-HT7 receptor is critically involved in acute and chronic inflammation of the gastrointestinal tract. Inflamm Bowel Dis.

[11] Ooi CJ, Makharia GK, Hilmi I (2016). Asia Pacific Consensus Statements on Crohn’s disease. Part 1: Definition, diagnosis, and epidemiology: (Asia Pacific Crohn’s Disease Consensus–Part 1). J Gastroenterol Hepatol.

[12] Ooi CJ, Makharia GK, Hilmi I (2016). Asia-Pacific consensus statements on Crohn’s disease. Part 2: Management. J Gastroenterol Hepatol.

[13] Enriksen M, Jahnsen J, Lygren I (2008). C-reactive protein: a predictive factor and marker of inflammation in inflammatory bowel disease. Results from prospective population-based study. Gut.

[14] Daperno M, D’Haens G, Van Assche G (2004). Development and validation of a new, simplified endoscopic activity score for Crohn’s disease: the SES-CD. Gastrointest Endosc.

[15] Rimola J, Ordás I, Rodriguez S (2011). Magnetic resonance imaging for evaluation of Crohn’s disease: validation of parameters of severity and quantitative index of activity. Inflamm Bowel Dis.

[16] Cátia A, Tiago CG, Francisca DC (2018). Clinical Course in Crohn’s Disease: Factors Associated with Behaviour Change and Surgery. Scand J Gastroenterol.

[17] Allison B, Alexandra S, Camila M (2019). Inflammatory Bowel Disease and the Risk for Cardiovascular Disease: Does All Inflammation Lead to Heart Disease?. Trends Cardiovasc Med.

[18] Tomasz B, Piotr E, Grażyna R (2019). Statement of the Expert Group on the Current Practice and Prospects for the Treatment of Complex Perirectal Fistulas in the Course of Crohn’s Disease. Pol Przegl Chir.

[19] Latorre R, Sternini C, De Giorgio R (2016). Enteroendocrine cells: a review of their role in brain-gut communication. Neurogastroenterol Motil.

[20] Md SS, Usha C, Salman A (2019). Characterization of Serotonin Signaling Components in Patients with Inflammatory Bowel Disease. J Can Assoc Gastroenterol.

[21] Shajib MS, Khan WI (2015). The role of serotonin and its receptors in activation of immune responses and inflammation. Acta Physiol (Oxf).

[22] Marianna C, Floriana V, Carla P (2020). Role of the Serotonin Receptor 7 in Brain Plasticity: From Development to Disease. Int J Mol Sci.

[23] Kim JJ, Bridle BW, Ghia JE (2013). Targeted Inhibition of Serotonin Type 7 (5-HT7) Receptor Function Modulates Immune Responses and Reduces the Severity of Intestinal Inflammation. J Immunol.

[24] Guseva D, Holst K, Kaune B (2014). Serotonin 5-HT7 Receptor Is Critically Involved in Acute and Chronic Inflammation of the Gastrointestinal Tract. Inflamm Bowel Dis.

[25] Andrea SP, Michael HS, Christopher JP (2009). Expression and Function of 5-HT7 Receptors in Smooth Muscle Preparations from Equine Duodenum, Ileum, and Pelvic Flexure. Res Vet Sci.

[26] Li Y, Chen B, Gao X, et al.Current Diagnosis and Management of Crohn’s Disease in China: Results From a Multicenter Prospective Disease Registry. BMC Gastroenterol. 201919:145. 10.1186/s12876-019-1057-2 PMC6697932 31420025

[27] James DL, Paul R, Brian GF (2020). Correlation of Stool Frequency and Abdominal Pain Measures With Simple Endoscopic Score for Crohn’s Disease. Inflamm Bowel Dis.

[28] Ingrid O, Jordi, Ignacio A (2019). Development and Validation of a Simplified Magnetic Resonance Index of Activity for Crohn’s Disease. Gastroenterology.

[29] Alexander SS, Vikesh K, Venkat SK (2019). Magnetic Resonance Enterography, Colonoscopy, and Fecal Calprotectin Correlate in Colonic Crohn’s Disease. BMC Gastroenterol.

[30] Cristina SF, Marta AB, Eduardo MA (2019). The Use of Serum Calprotectin as a Biomarker for Inflammatory Activity in Inflammatory Bowel Disease. Rev Esp Enferm Dig.

